# A Rare Case of Undifferentiated Carcinoma of the Colon with Rhabdoid Features: A Case Report and Review of the Literature

**DOI:** 10.1155/2015/531348

**Published:** 2015-04-30

**Authors:** E. Moussaly, J. P. Atallah

**Affiliations:** Staten Island University Hospital, 475 Seaview Avenue, Staten Island, NY 10305, USA

## Abstract

Malignant rhabdoid tumors were originally described in children. Subsequently, the same histological pattern was described in adults. Malignant rhabdoid tumors are aggressive neoplasms that have been reported in multiple organs. To our best knowledge, only 16 previous cases of rhabdoid tumor in the colon have been described in the literature. We present the case of an 87-year-old lady who was diagnosed with a rhabdoid tumor of the colon that relapsed rapidly after surgical resection. The literature concerning this unusual neoplasm was subsequently reviewed with comparison of all known cases in the literature.

## 1. Introduction

In 1978, Beckwith and Palmer first described Wilms' tumors with “rhabdomyosarcomatoid” features in children. This pattern was characterized by diffuse sheets of polygonal cells with acidophilic cytoplasm and rounded vesicular nuclei [1]. Subsequently, this pattern was described in adults and in multiple extrarenal locations such as the gastrointestinal tract, urinary tract, skin, and central nervous system and termed malignant rhabdoid tumors (MRT) [2, 3]. These malignant rhabdoid tumors can be pure or mixed with other malignancies. The existence of MRT as a distinct oncological entity is still under debate [4, 5]. MRT are invariably associated with unfavorable prognosis [2, 3]. The first case of MRT in the colon was described in 1994 by Yang et al. [6]. Since this publication, 15 cases of malignant colon cancer with rhabdoid features have been published in the literature to our knowledge [5–19]. We present the case of an 87-year-old female who was complaining of abdominal pain and was found to have undifferentiated carcinoma of the transverse colon with rhabdoid features.

## 2. Case Presentation

The patient was an 87-year-old Caucasian female of Russian descent who presented to our hospital with a two-day history of generalized weakness, profuse nonbilious nonbloody vomiting, and decreased oral intake. The patient complained of diffuse abdominal pain but had no change in bowel movement. The patient had been suffering from decreased appetite for weeks. The patient's past medical and surgical history are noncontributory because the patient had not seen a physician nor taken a medication in many years. In the emergency department, vital signs showed blood pressure of 88/54, pulse rate of 115 bpm, a respiratory rate of 20, and a temperature of 98.8 F. Physical examinations showed a frail looking lady with pale conjunctiva and skin, lungs were clear to auscultation bilaterally, and heart auscultation did not elicit any murmur rubs or gallop. Abdominal examination elicited a right upper quadrant mass on deep palpation without associated tenderness or distention and with positive bowel sounds in all quadrants. The rest of the physical exam was unremarkable. A stool guaiac test performed at bedside was negative. On further interrogation of the family, we were informed that the patient was a nonsmoker and occasional alcohol user and had no known drug allergies. The patient had no family history of malignancy.

The patient was urgently stabilized in the emergency department with intravenous fluids, transfusion therapy, and antibiotics and was admitted to the intensive care unit for monitoring.

Blood tests on admission were as shown in Table 1.

An emergent CT scan of the abdomen and pelvis done without contrast showed a large concentric and enveloping 12.1 × 9.3 × 8.4 cm mass along the hepatic flexure of the transverse colon with associated mass-effect and obstruction along a portion of the distal jejunum and innumerable splenic hypodensities. A chest radiograph done on admission did not show any suspicious nodules. A colonoscopy showed a friable, infiltrative, and ulcerated tumor which occupied 100% of the circumference of the transverse colon and caused a severe 80% obstruction of the lumen. Biopsies were taken. Additional findings included diverticulosis in the sigmoid and descending colon and incidental polyps which were removed. The patient underwent right hemicolectomy, partial omentectomy, small bowel resection with primary anastomosis, and a twelve-lymph node resection. The postoperative course was unremarkable. Macroscopically, the mass was a 12 × 9 × 9 cm necrotic tumor. Pathological findings showed a high grade malignant neoplasm, in favor of undifferentiated carcinoma with rhabdoid features involving ulcerated mucosal and submucosal fragments with extensive necrosis. The tumor was positive for calretinin, pancytokeratin, vimentin, CAM 5.2, and neuron specific enolase which are in favor of the aforementioned diagnosis. The tumor was focally positive for epithelial membrane antigen. One of twelve lymph nodes that were found in the resected specimen was positive for micrometastasis. The tumor extended to the free serosal surface of the right colon and a loop of small bowel with extensive vascular involvement and no perineural involvement. Surgical margins were negative microscopically. The final pathologic stage was pT4B, pN1 (mic), and pMx with microsatellite instability. The initial staging was stage IIIC without ruling out metastasis since the splenic masses were never evaluated with pathology. Upon follow-up with the oncologist and further discussion of the case with the family, a trial of capecitabine was suggested. The family opted not to undergo any treatment but decided to follow with the oncologist closely and manage symptoms.

Upon follow-up with the oncologist one month after the initial assessment, a repeat CT of the abdomen showed development of moderate amount of intra-abdominal pelvic ascites and interval development of 6.1 × 3.1 cm heterogenous mass in the right abdominal wall consistent with tumor recurrence associated with new mesenteric, gastrohepatic ligament and retropritoneal adenoapthies. Multiple low-density splenic lesions stable in appearance from the previous study were identified. A repeat CT of the chest showed a 1.2 cm solid nodule in the left lung apex. Physical exam in the clinic elicited effectively a 5 cm nontender palpable mass at the surgical scar. Repeat CEA was 11. The patient passed away two months later from complications of her disease.

## 3. Discussion

Colon carcinoma with rhabdoid features is an uncommon pathological entity. It is almost always associated with unfavorable prognosis. The existence of this tumor as an independent pathological entity is still under debate since it is considered by some experts to be merely a phenotypical variation of the tumor [4]. Malignant rhabdoid tumors have been described in a variety of organs. They share common histology and immunophenotype and can be mixed with different types of neoplasms (carcinomas, melanomas, and sarcomas). These tumors are described as “composite” when the rhabdoid phenotype is mixed with another type of identifiable neoplasm and termed “pure” when the rhabdoid features are the only identifiable phenotype. Histologically, the rhabdoid phenotype is characterized by the presence of pleomorphic cells with large, eccentric nuclei, prominent nucleoli, abundant and eosinophilic cytoplasm, paranuclear inclusions of intermediate filaments, and abundant mitotic figures [7]. Cytokeratin and vimentin are frequently found on immunochemistry [8].

Table 2 shows the clinical characteristics of all the cases of poorly differentiated carcinoma of the colon with rhabdoid features that we found in the literature [5–19]. This entity seems to be a disease of the elderly with a mean age of 70 years at presentation. It is equally distributed between both sexes with 9 males and 8 females. Almost all patients presented with abdominal symptoms including abdominal pain, abdominal mass, and gastrointestinal bleed. This is quite an unusual presentation of colon cancer that tends to have more occult presentation and could be a reflection of the aggressive nature of this type of tumor. In fact, the average size at presentation, which we calculated based on the tumor's longest diameter, was 8.8 cm. The largest tumor is 12 cm and the smallest one is 3 cm. These tumors were distributed equally along the colon. Tumors were described from the cecum all the way to the rectum. Six of the patients had identifiable metastasis on presentation. Twelve patients had at least one positive lymph node invasion, 2 were negative, and 3 were not specified. These facts also reflect the aggressive nature of this particular phenotype. Nine patients had composite rhabdoid tumors of the colon, 6 had pure rhabdoid tumors, and one case was not specified. The overall survival from this tumor even after surgical intervention seems to be unfavorable with a large majority of the patients surviving for less than six months even after surgery. Only three patients were documented to have received chemotherapy. One patient received 12 cycles of FOLFOX and was still alive at the time the paper was written surviving 36 months [8]. Another received a trial of capecitabine and oxaliplatin with a survival of 6 months [9]. The third patient received a trial of bevacizumab and cetuximab and had an 8-month survival [10]. The uncommon occurrence of malignant extrarenal rhabdoid tumors (MERT) has made it complicated to establish adequate survival-improving protocols. Horazdovsky et al. concluded in their meta-analysis that surgery and actinomycin might improve survival in MERT [20].

Imaging does not seem to be particularly helpful in diagnosing tumors with rhabdoid features since it does not present any specific radiological findings [21]. The rhabdoid phenotype is characterized in pathology by the presence of pleomorphic cells with large eccentric nuclei, prominent nucleoli, a large eosinophilic cytoplasm, and intermediate filament inclusions. Cytokeratin and vimentin are found regularly on immunochemistry, although in variable degrees [22]. Other markers described in the rhabdoid phenotype are epithelial membrane antigen, CAM5.2, CD99, synaptophysin, and neuron specific enolase. Some tumors express the p53 gene mutation and others show microsatellite instability [8]. Since a wide variety of tumors may exhibit rhabdoid features, the WHO recommends, in its classification of soft tumors, that the rhabdoid phenotype be reflected in the final pathological diagnosis, either as composite extrarenal rhabdoid tumor (if the tumor appears to be a mix of rhabdoid and non-rhabdoid elements) or as a modifier (if the rhabdoid phenotype is more diffuse in the tumor) [23].

In 1998, Versteege et al. described* SMARCB1* biallelic inactivation in rhabdoid tumors [24, 25].* SMARCB1* (also known as BAF47) is a core subunit of the SWI/SNF chromatin remodeling complex which regulates chromatin structure and thus plays an important role in gene expression [26]. The inactivation of other SWI/SNF subunits such as the BRM gene has also been implicated in rhabdoid tumor oncogenesis [25]. The SWI/SNF chromatin remodeling complex has been hypothesized to play a key role in the formation of a variety of neoplasms [27]. Even though rhabdoid tumors are aggressive tumors, they present a simple genetic configuration with lack of chromosomal instability and a mutation rate among the lowest in all sequenced cancer genomes with loss of* SMARCB1* as the only consistently recurrent event [28, 29]. Up to one-third of patients with rhabdoid tumors have been found to have a genetic predisposition to these tumors secondary to a germline* SMARCB1* alteration [30]. Germline* SMARCB1* mutations strongly predispose to rhabdoid tumors [31] and subsequent somatic loss of the other allele can lead to cancer formation.* SMARCB1* biallelic loss is now used in diagnosing these tumors by detecting loss of protein expression in the nucleus by immunostaining [32]. Thus atypical teratoid/rhabdoid tumors (central nervous system rhabdoid tumors) and renal and extrarenal rhabdoid tumors seem to be genetically related and* SMARCB1* loss detection can thus play an integral part in differentiating rhabdoid tumors from other tumors with similar histologic features [33]. This low mutation rate could imply that even limited genetic disturbances can drive cancer formation. This argument is also supported by the fact that other cancers also have low mutation rates such as retinoblastoma, neuroblastoma, and AML [34]. The oncogenic properties of* SMARCB1* loss seem to be related to its effect on p16 INK4a and cyclin D1 which play a significant role in the cell cycle and on RhoA signaling which enhances cell migration [33]. Understanding the epigenetic consequences of* SMARCB1* loss might provide insight to oncogenesis and may help in developing therapies for a multitude of SWI/SNF mutant neoplasms [34].* SMARCB1* losses have also been described in other tumors such as renal medullary carcinoma, epithelioid sarcomas, and nerve sheath neoplasms [33].

## 4. Conclusion

Tumors with rhabdoid features have been described in multiple organs. The rhabdoid feature seems to be associated with poor prognosis. We present the case of an 87-year-old female who was found to have an undifferentiated carcinoma of the colon with rhabdoid features. To our best knowledge, this case is the seventeenth case of a tumor with rhabdoid phenotype to be described in the colon.

## Figures and Tables

**Table 1:** 

Sodium	130 mmol/L
Chloride	90 mmol/L
Potassium	3.8 mmol/L
Bicarbonate	20 mmol/L
Creatinine	1.83 mg/dL
BUN	51 mg/dL
Lactic acid	1.5 mmol/L
Glucose	175 mg/dL
Magnesium	2.9 mg/dL
ALT	45 IU/L
AST	20 IU/L
Alkaline phosphatase	95 IU/L
Total bilirubin	0.7 mg/dL
Albumin	2.8 g/dL
Lipase	14 U/L
WBC	14.54 TH/mm3
Hb	6.3 g/dL
MCV	75.3 MCM3
RDW	14.2%
Platelets	576 TH/mm3
Segmented neutrophils	74%
Lymphocytes	1%
Band neutrophils	19%
CEA	4.3

**Table 2:** Clinical characteristics of all cases of poorly differentiated carcinoma of the colon with rhabdoid features described in the literature, to our knowledge. Age is in years. Size means the largest diameter of the tumor in centimeters. *DNS: did not specify*. [5–19].

	Age	Sex	Size	Location	Presentation	Metastasis	Type	Survival
Baba et al.	45	F	—	—	Abdominal pain	—	—	6 weeks
Romera Barba et al.	77	M	*DNS *	Descending colon	Abdominal pain	No	Pure	2 months
Lee et al. case 1	62	M	4.5	Sigmoid colon	Occult blood in stool	No	Composite	36 months still alive
Lee et al. case 2	83	M	6.5	Rectum	Rectal mass	Yes	Composite	one month
Remo et al.	73	F	10	Right colon	Rectal bleed	No	Composite	6 months
Pancione et al.	71	F	10	Right colon	Abdominal pain	Yes	Pure	8 months
Nakamura et al.	76	M	14	Cecum	Abdominal pain	Yes	Pure	3 months
Marcus et al.	84	F	7	Transverse colon	Abdominal mass	No	Composite	12 months still alive
Yang et al.	75	M	15	Transverse colon	*DNS *	No	Pure	2 weeks
Agaimy et al.	79	M	9	Cecum	—	No	Composite	6 months
Mastoraki et al.	62	F	10	Descending colon	Abdominal pain	Yes	Pure	4 months
Lee et al.	63	M	3	Right colon	Weakness	Yes	Pure	*DNS *
Chetty and Bhathal	72	F	6	Cecum	Abdominal mass	Yes	Composite	*DNS *
Kono et al.	66	M	13	Cecum	Abdominal mass	No	Composite	6 weeks
Oh et al.	69	F	3.5	Sigmoid colon	Blood in stools	No	Composite	6 months
Macák and Kodet	50	M	—	Rectum	—	—	Composite	—
Our case	87	F	12	Transverse colon	Abdominal mass	No	Composite	2 months

## References

[1] Beckwith J. B., Palmer N. F. (1978). Histopathology and prognosis of Wilms tumor: results from the first national Wilms' tumor study. *Cancer*.

[2] Parham D. M., Weeks D. A., Beckwith J. B. (1994). The clinicopathologic spectrum of putative extrarenal rhabdoid tumors: an analysis of 42 cases studied with immunohistochemistry or electron microscopy. *The American Journal of Surgical Pathology*.

[3] Wick M. R., Ritter J. H., Dehner L. P. (1995). Malignant rhabdoid tumors: a clinicopathologic review and conceptual discussion. *Seminars in Diagnostic Pathology*.

[4] Weeks D. A., Beckwith J. B., Mierau G. W. (1989). Rhabdoid tumor: an entity or a phenotype?. *Archives of Pathology and Laboratory Medicine*.

[5] Chetty R., Bhathal P. S. (1993). Caecal adenocarcinoma with rhabdoid phenotype: an immunohistochemical and ultrastructural analysis. *Virchows Archiv—A Pathological Anatomy and Histopathology*.

[6] Yang A. H., Chen W. Y. K., Chiang H. (1994). Malignant rhabdoid tumour of colon. *Histopathology*.

[7] Romera Barba E., Sánchez Pérez A., Duque Pérez C., García Marcilla J. A., Vázquez Rojas J. L. (2014). Malignant rhabdoid tumor of the colon: a case report. *Cirugia Espanola*.

[8] Lee S. H., Seol H., Kim W. Y. (2013). Rhabdoid colorectal carcinomas: reports of two cases. *Korean Journal of Pathology*.

[9] Remo A., Zanella C., Molinari E. (2012). Rhabdoid carcinoma of the colon: a distinct entity with a very aggressive behavior: a case report associated with a polyposis coli and review of the literature. *International Journal of Surgical Pathology*.

[10] Pancione M., Di Blasi A., Sabatino L. (2011). A novel case of rhabdoid colon carcinoma associated with a positive CpG island methylator phenotype and BRAF mutation. *Human Pathology*.

[11] Baba Y., Uchiyama T., Hamada K. (2014). A case report of undifferentiated carcinoma of the sigmoid colon with rhabdoid features. *Nihon Shokakibyo Gakkai Zasshi*.

[12] Nakamura I., Nakano K., Nakayama K. (1999). Malignant rhabdoid tumor of the colon: report of a case. *Surgery Today*.

[13] Marcus V. A., Viloria J., Owen D., Tsao M.-S. (1996). Malignant rhabdoid tumor of the colon. Report of a case with molecular analysis. *Diseases of the Colon & Rectum*.

[14] Agaimy A., Rau T. T., Hartmann A., Stoehr R. (2014). SMARCB1 (INI1)-negative rhabdoid carcinomas of the gastrointestinal tract: clinicopathologic and molecular study of a highly aggressive variant with literature review. *American Journal of Surgical Pathology*.

[15] Mastoraki A., Kotsilianou O., Papanikolaou I. S., Foukas P. G., Sakorafas G., Safioleas M. (2009). Malignant rhabdoid tumor of the large intestine. *International Journal of Colorectal Disease*.

[16] Lee S. J., Kim T. H., Ko D. H. (2010). Undifferentiated adenocarcinoma of the colon with rhabdoid features. *Korean Journal of Gastrointestinal Endoscopy*.

[17] Kono T., Imai Y., Imura J. (2007). Cecal adenocarcinoma with prominent rhabdoid feature: report of a case with immunohistochemical, ultrastructural, and molecular analyses. *International Journal of Surgical Pathology*.

[18] Oh H.-K., Cho C.-H., Kum Y.-S. (2008). Adenocarcinoma of the sigmoid colon with prominent rhabdoid features—a case report. *Korean Journal of Pathology*.

[19] Macák J., Kodet R. (1995). Rectal adenocarcinoma with rhabdoid phenotype. *Pathologica*.

[20] Horazdovsky R., Manivel J. C., Cheng E. Y. (2013). Surgery and actinomycin improve survival in malignant rhabdoid tumor. *Sarcoma*.

[21] Roebuck D. J. (1996). The role of imaging in renal and extra-renal rhabdoid tumours. *Australasian Radiology*.

[22] Voglino C., Scheiterle M., Di Mare G. (2014). Malignant rhabdoid tumor of the small intestine in adults: a brief review of the literature and report of a case. *Surgery Today*.

[23] Fletcher C., Unni K., Mertens F. (2002). *Pathology and Genetics of Tumours of Soft Tissue and Bone*.

[24] Versteege I., Sévenet N., Lange J. (1998). Truncating mutations of hSNF5/INI1 in aggressive paediatric cancer. *Nature*.

[25] Kahali B., Yu J., Stefanie B. (2014). The silencing of the SWI/SNF subunit and anticancer gene BRM in Rhabdoid tumors. *Oncotarget*.

[26] Phelan M. L., Sif S., Narlikar G. J., Kingston R. E. (1999). Reconstitution of a core chromatin remodeling complex from SWI/SNF subunits. *Molecular Cell*.

[27] Wilson B. G., Roberts C. W. M. (2011). SWI/SNF nucleosome remodellers and cancer. *Nature Reviews Cancer*.

[28] Lee R. S., Stewart C., Carter S. L. (2012). A remarkably simple genome underlies highly malignant pediatric rhabdoid cancers. *The Journal of Clinical Investigation*.

[29] Jackson E. M., Sievert A. J., Gai X. (2009). Genomic analysis using high-density single nucleotide polymorphism-based oligonucleotide arrays and multiplex ligation-dependent probe amplification provides a comprehensive analysis of INI1/SMARCB1 in malignant rhabdoid tumors. *Clinical Cancer Research*.

[30] Sévenet N., Sheridan E., Amram D., Schneider P., Handgretinger R., Delattre O. (1999). Constitutional mutations of the hSNF5/INI1 gene predispose to a variety of cancers. *The American Journal of Human Genetics*.

[31] Biegel J. A., Kalpana G., Knudsen E. S. (2002). The role of INI1 and the SWI/SNF complex in the development of rhabdoid tumors: meeting summary from the workshop on childhood atypical teratoid/rhabdoid tumors. *Cancer Research*.

[32] Lee R. S., Roberts C. W. M. (2013). Rhabdoid tumors: an initial clue to the role of chromatin remodeling in cancer. *Brain Pathology*.

[33] Roberts C. W. M., Biegel J. A. (2009). The role of SMARCB1/INI1 in development of rhabdoid tumor. *Cancer Biology & Therapy*.

[34] Eaton K. W., Tooke L. S., Wainwright L. M., Judkins A. R., Biegel J. A. (2011). *Spectrum of SMARCB1/INI1 mutations in familial and sporadic rhabdoid tumors [Ph.D. thesis]*.

